# The emerging role of high-intensity focused ultrasound (HIFU) as a symptomatic treatment for Parkinson's disease and essential tremor: a narrative review

**DOI:** 10.1055/s-0046-1827056

**Published:** 2026-08-13

**Authors:** Karina Silveira Massruha, Ananda Carolina Moraes de Falcone, Isadora Santos Ferreira, Jacy Bezerra Parmera, Polyana Vulcano de Toledo Piza, Rodrigo Gobbo Garcia, Ellison Fernando Cardoso, Luis Filipe de Souza Godoy, Stephany Tueini Toogood, Anna Verena de Carvalho Leite, Rubens Gisbert Cury

**Affiliations:** 1 Universidade de São Paulo, Faculdade de Medicina, Departamento de Neurologia, Centro de Distúrbios do Movimento, São Paulo SP, Brazil.; 2 Einstein Hospital Israelita, Departamento de Neurologia, São Paulo SP, Brazil.; 3 Einstein Hospital Israelita, Departamento de Imagem, São Paulo SP, Brazil.; 4 Einstein Hospital Israelita, Centro de Medicina Intervencionista, São Paulo SP, Brazil.; 5 Universidade de São Paulo, Faculdade de Medicina, Hospital das Clínicas, Instituto de Radiologia, Departamento de Neurorradiologia, São Paulo SP, Brazil.

**Keywords:** Essential Tremor, Parkinson Disease, High-Intensity Focused Ultrasound Ablation, Tremor, Movement Disorders

## Abstract

The high-intensity focused ultrasound (HIFU) technique has become precise, incisionless, and ablative for treating essential tremor (ET) and Parkinson's disease (PD), driven by advances in magnetic resonance imaging (MRI) thermometry and tractography. The current review integrates the main clinical outcomes associated with established and emerging HIFU targets, synthesizing efficacy data, adverse effect (AE) patterns, and procedural characteristics relevant to clinical decision-making. In ET, ventral intermediate nucleus (VIM) thalamotomy results in tremor reductions ranging from 56 to 81%, with a long-term sustained benefit of approximately 73%. In tremor-dominant PD (TdPD), VIM ablation achieves tremor suppression of approximately 62%. Comparatively, HIFU-guided subthalamotomy provides broader motor benefits, with 53 to 57.8% improvement in the Movement Disorder Society's Unified Parkinson's Disease Rating Scale, Part III (MDS-UPDRS III), as well as significant reductions in rigidity and bradykinesia. Pallidotomy improves “off” state motor symptoms and dyskinesias, whereas pallidothalamic tractotomy leads to 52% global motor improvement and 91% tremor reduction. The AEs vary according to the targets. In VIM procedures, gait imbalance (16–40%) and sensory disturbances (9–26%) predominate, along with dysarthria and dysmetria. Lesions to the subthalamic nucleus (STN) are most commonly associated with dysarthria (10–26%) and gait instability (15–48%). Procedures targeting the globus pallidus internus (GPi) or pallidothalamic tract (PTT) may also present with gait instability or dysarthria, which are generally mild and transient. Although the SDR has historically limited eligibility, recent evidence demonstrates that patients with SDR ≤ 0.40 may still experience meaningful improvement, supporting more individualized selection. In the current therapeutic landscape, HIFU offers immediate clinical benefits, absence of implanted hardware, and simplified recovery, positioning it as an alternative or complementary therapy to deep brain stimulation (DBS).

## INTRODUCTION

High-intensity focused ultrasound (HIFU) has emerged as an unprecedented modality for treating neurological disorders. This novel, minimally invasive, and incision-free approach offers a new strategy for addressing a range of brain diseases and has witnessed rapid advancements in both research and clinical applications, particularly in the management of movement disorders and other functional neurological disorders. 1

The exploration of focused ultrasonic waves for biological applications dates back to 1942, when Lynn et al. first investigated their potential. 2 The foundational work of Fry et al. in the 1950s further advanced the field, as they developed focused ultrasound technology for both clinical and research purposes. 3 In addition to the advent of levodopa therapy in the 1960s, technology was constrained by the necessity of performing craniotomy to effectively transmit ultrasound energy to the target. All these factors led to the procedure being considered invasive and subsequently abandoned. Decades later, technological advances enabled transcranial sonication through an intact human skull, rendering it a minimally invasive procedure.

Recently, computed tomography (CT) has been used to address the challenges posed by skull thickness and density, thereby minimizing ultrasound deflection and absorption. 4 The integration of focused ultrasound technology with real-time magnetic resonance imaging (MRI) thermography enabled precise thermal lesioning through intact skull transmission. 1 5 6

In 2009, Martin et al. demonstrated the first successful clinical application of HIFU for the treatment of therapy-resistant neuropathic pain by targeting the posterior part of the thalamic central lateral nucleus. 5 A few years later, Elias et al. reported the first series of 15 medication-refractory essential tremor (ET) patients undergoing HIFU thalamotomy, revealing significant improvement in tremor of the contralateral hand. 1

Based on two multicenter, randomized, sham-controlled clinical trials with positive outcomes, 7 8 the United States Food and Drug Administration (FDA) approved HIFU for the treatment of ET in 2016. Subsequently, in 2018, it received FDA approval for the management of tremor-dominant Parkinson's disease (TdPD) in adults. These studies underscore the potential of HIFU and provide compelling initial evidence of its safety, reliability, and precision. To date, several other clinical trials have been published demonstrating the benefits and safety of its treatment, most targeting the ventral intermediate nucleus (VIM), 9 10 11 12 13 14 15 16 17 the subthalamic nucleus (STN) for TdPD, 18 19 20 21 and, more recently, the globus pallidus internus (GPi) 22 23 and pallidothalamic tract (PTT). 24

In this narrative review, we examined the indication criteria and outcomes of HIFU for ET and Parkinson's disease (PD), the main targets, and the debate between HIFU and deep brain stimulation (DBS).

## METHODS

This narrative review was based on a structured search of the medical literature to identify studies evaluating the use of HIFU in the treatment of ET and PD. Electronic databases including PubMed/MEDLINE, Scopus, and Web of Science were searched for relevant publications. The most recent search was conducted in December 2025.

Search terms included combinations of keywords such as *focused ultrasound* , *MR-guided focused ultrasound* , *HIFU* , *essential tremor* , *Parkinson's disease* , *VIM thalamotomy* , *subthalamotomy* , *pallidotomy* , and *pallidothalamic tractotomy* .

Original clinical studies, prospective and retrospective cohorts, randomized trials, and relevant meta-analyses evaluating the clinical outcomes, safety profile, and technical aspects of HIFU were considered. Studies focusing on ET and PD, particularly those addressing clinical outcomes, anatomical targets, and comparisons with DBS, were prioritized. The reference lists of selected articles were also reviewed to identify additional relevant studies.

## HIGH-INTENSITY FOCUSED ULTRASOUND

The minimally-invasive HIFU technique operates under closed-loop image guidance, allowing submillimeter precision in the creation of thermal lesions. During the procedure, patients remain awake, enabling real-time clinical assessments that facilitate accurate lesion placement. 25

Currently, HIFU requires the use of a stereotactic frame attached to the patient's shaved head after local anesthesia. Researchers are still exploring the possibility of performing the procedure without head shaving. 26 27 The patients are placed on an MRI table in the supine position with their heads connected to a focused ultrasound transducer. Then, T2-weighted spin-echo images are acquired using a 3-Tesla MRI scanner.

Additionally, previous anatomical MRI and CT scans are combined to correct for variations in skull thickness and density, which are essential for achieving precise heating and targeting. A subset of patients has skull density characteristics that prevent sufficient heating of the intracranial structures during HIFU, which can often be predicted by calculating the skull density ratio (SDR) before treatment. An ideal SDR is considered > 0.40, and low rates are typically considered poor candidates, especially < 0.35. 6

Nevertheless, recent evidence suggests that clinical outcomes may not differ substantially across SDR ranges. A systematic review and meta-analysis evaluating HIFU thalamotomy outcomes across SDR thresholds found that patients with a low SDR (≤ 0.40) experienced tremor improvement comparable to those with SDR > 0.40. Pooled data demonstrated sustained clinical efficacy at both 3 and 12 months, with no statistically significant difference between groups (standardized mean difference [SMD]: −0.02; 95%CI: −0.46–0.42). 28

Similarly, a large case-matched cohort study including 270 patients demonstrated that individuals with SDR < 0.40 can be safely and effectively treated with HIFU thalamotomy. Tremor improvement was comparable between low and high SDR groups at all postoperative time points. 29 In line with the expanding evidence base, recent regulatory updates now support FDA eligibility for patients with SDR values as low as 0.30, reflecting accumulating longitudinal data demonstrating maintained safety and efficacy in selected low-SDR candidates. 30 31

**Figure 1:**
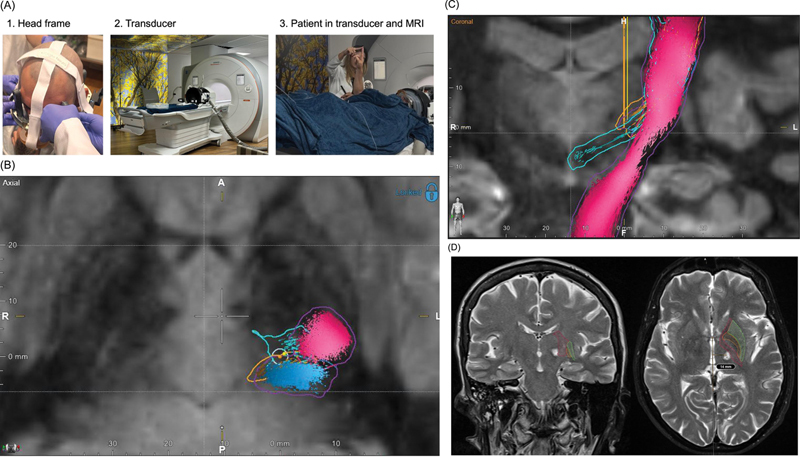
Abbreviations: DRTT, dentato-rubro-thalamic tracts; ET, essential tremor; FGATIR, fast gray matter acquisition t1 inversion recovery; HIFU, high-intensity focused ultrasound; MRI, magnetic resonance imaging; VIM, ventral intermediate nucleus of the thalamus. Workflow and imaging stages of HIFU thalamotomy for ET. ( **A** ) Preparation phase: stepwise setup including 1. head frame fixation, 2. positioning of the transducer within the MRI environment, and 3. patient alignment and intraoperative clinical testing. ( **B** ) FGATIR-MRI with diffusion tractography illustrating HIFU targeting of the VIM for tremors. Axial FGATIR image showing the two planned ablation targets (Trajectory 1–orange sphere; Trajectory 2–yellow sphere) within the VIM. The surrounding white matter tracts are displayed for anatomical reference: the internal capsule (pink) is located laterally, while the medial lemniscus (blue) lies posteromedially to the target. The DRTTs are also delineated, with the nondecussating DRTT in orange and the decussating DRTT in green. ( **C** ) Coronal FGATIR view demonstrating the vertical alignment of both trajectories corresponding to the first and second sonications. The sequence provides excellent gray–white matter contrast, enabling precise visualization between the lesion target and its spatial relationship to adjacent fiber pathways. ( **D** ) Postprocedure MRI showing the resulting lesion in the left thalamus (VIM), located approximately 14 mm lateral to the midline. Although in this case the target coincided with the standard stereotactic coordinates typically used for thalamotomy, this is not true for most patients. Therefore, detailed imaging analysis becomes essential for assessing individual anatomy and ensuring the safety and precision of the procedure. The lesion's core and surrounding edema are visible, with neighboring tracts identified for spatial reference: internal capsule (pink, lateral), medial lemniscus (blue, posteromedial), as well as nondecussating (orange) and decussating (green) DRTT.

During the procedure, low-energy sonication is used to gradually raise the temperature up to 40 to 45 °C, while MRI thermography is used to confirm the accuracy of the target. By applying sublesional temperatures, temporary physiological effects can be produced, likely by inactivating neural elements such as thalamic neurons, which helps map the precise target and reduce the risk of adverse effects (AEs) from misdirected lesions. 25 After each sonication, patients are monitored using MRI-based thermometry and evaluated for tremor reduction and potential side effects. Once the target is confirmed, therapeutic sonication is administered with increased intensity to achieve tissue ablation at temperatures between 55 and 60 °C. 32 Following the procedure, the transducer and head frame are removed, clinical evaluation is performed, a new MRI scan is acquired, and patients are typically discharged after 2 to 3 hours of hospital observation. The preparation stage is shown in Figure 1 .

## WHO ARE THE CANDIDATES?

Treatments involving HIFU in movement disorders are intended for patients diagnosed with medication-refractory ET, 7 TdPD, 8 and for the treatment of PD symptoms such as bradykinesia, rigidity, and dyskinesia. 22

### Essential tremor

The most common movement disorder in adults, ET has an overall prevalence of approximately 1.3 and 4.8% among those older than 65 years. 33 Approximately 50% of patients with ET are considered medication-refractory, 33 which means that they have tried at least two first-line therapy medications in adequate dosages (i.e. primidone, β blockers) and found them to be insufficient for tremor control. Neurologists should consider these patients as candidates for alternative therapies, such as DBS or HIFU, according to their specific profiles.

Since VIM is considered the primary thalamic relay between the cerebellum and motor cortex, it is acknowledged as the preferred target for tremors. 34

Although tremors in ET are generally symmetrical, one side often predominates, leading to significant functional impairment and reduced quality of life (QoL). 35 Initially, thalamotomy was approved only for unilateral treatment; however, staged bilateral HIFU was later authorized for ET patients, supported by safety data demonstrating its feasibility when performed at least 9 months apart. 17

### Parkinson's disease

As a neurodegenerative disorder, PD has doubled in prevalence over the past generation and currently affects over 6 million people worldwide. 36 It is pathologically characterized by the progressive loss of dopaminergic cells in the Substantia Nigra pars compacta (SNpc) of the midbrain and the presence of Lewy bodies throughout the central nervous system. 37 38 As it is a chronic disease, every neurologist eventually faces treatment challenges in overcoming motor complications during its moderate and advanced stages. Alternative therapies can be offered to provide a better QoL and improvement of symptoms when patients present with motor fluctuation, dyskinesia, levodopa (L-Dopa)-refractory tremor, and L-Dopa intolerance.

Patients with TdPD with asymmetric tremor impairment were initially treated with HIFU targeting the VIM, with studies demonstrating both immediate and sustained improvements postprocedure, usually measured by the Unified Parkinson's Disease Rating Scale, Part III (UPDRS III) motor examination score. 8 9 15 39

Patients with PD and asymmetric motor symptoms who are either unsuitable or unwilling to undergo DBS have demonstrated positive outcomes with HIFU targeting the STN and, more recently, the PTT. 24 This approach has shown significant improvements in the core motor symptoms of PD, including rigidity, bradykinesia, and tremors. 18 19 20 21

Additionally, HIFU targeting the GPi has proven beneficial for patients with PD experiencing troublesome dyskinesias, motor fluctuations, and motor impairment during “off-medication” states. 22 23

### Other types of tremors

A single-blinded, prospective case series 40 evaluated the safety and efficacy of unilateral HIFU thalamotomy targeting the VIM in six patients with medication-refractory tremors of non-ET etiologies, including three patients with PD-related tremor, two with tremor associated with dystonia (cervicobrachial dystonia and writer's cramp), and one with dystonia gene-associated tremor.

The primary outcome was the reduction in contralateral hemibody tremor severity, as measured by the lateralized tremor rating scale (TRS) Parts A and B. The mean composite TRS score improved by 42.2% at 1 week, 52.0% at 1 month, 55.9% at 3 months, and 52.9% at 6 months postoperatively. These improvements were statistically significant at all time points ( *p* < 0.05). Despite the small sample size and short follow-up duration, this study provided Class IV evidence supporting the feasibility, safety, and short-term efficacy of HIFU thalamotomy for non-ET tremor syndromes. 40

## CLINICAL OUTCOMES

### Essential tremor

#### *Short-term (up to 2 years)*

Initial pilot studies with HIFU thalamotomy, in 2013, demonstrated substantial improvement in contralateral hand tremor scores, ranging from 75 to 81%, 1 25 which enforced the investigation of this interval technique, rather than being followed by a multicenter, randomized, sham-controlled, clinical trial with 47% tremor improvement. 7 These data are complemented by more recent studies that have shown 56 to 80% tremor improvement 6 months after the procedure, suggesting a learning curve of the technique as a reason for higher rates of improvement. 13 16 41

Similar and robust effects within the first 6 months, with approximately 45% tremor improvement in the contralateral hand treated, were found in a recent meta-analysis. 42 Moreover, most studies have also evaluated the QoL in these cohorts, finding an improvement of approximately 41% at 6 months after the procedure. 42

Recent advancements have shown encouraging outcomes in the treatment of axial tremors affecting both the head and voice. Arcadi et al. observed a remarkable 100% axial tremor suppression in approximately 71% of the patient cohort at 6 months posttreatment. 16

Comparatively, staged bilateral HIFU thalamotomy for ET has shown efficacy and safety similar to unilateral treatment, and it has been FDA-approved since December 2022. 13 17 Treatment of tremor severity in the limbs contralateral to the second-side improved the symptom by approximately 45%, with sustained results at the 3-month follow-up. 13

A prospective, open-label, multicenter study by Kaplitt et al. involving 51 patients demonstrated that staged bilateral HIFU significantly improved tremor severity and functional disability. 17 At 3 months, the patients experienced a 66% improvement in tremor-motor scores and an 81% reduction in postural tremor scores, both of which were sustained at 6 and 12 months. Furthermore, the procedure proved effective in patients with head and voice tremors, with 67% of voice and 71% of head cases showing substantial treatment responses. Additionally, a 73% improvement in the Clinical Rating Scale for Tremor (CRST) Part C score was observed, reflecting an improvement in functional disability. These results confirm the sustained efficacy of HIFU in tremor control and QoL enhancement in patients with ET, as summarized in Table 1 .

**Table 1:** Short-term (≤ 2 years) clinical studies on HIFU thalamotomy targeting the VIM for ET

Year	Author	Study design	Indication	Target	Patients (n)	Follow-up	Tremor improvement (%)
2013	Elias et al. 1	Prospective, open-label, uncontrolled pilot	Medication-refractory ET	VIMunilateral	15	1 year	75
2013	Lipsman et al. 25	Prospective, open-label, uncontrolled pilot	Medication-refractory ET	VIMunilateral	4	3 months	81
2015	Chang et al. 6	Prospective, open-label, uncontrolled pilot	Medication-refractory ET	VIMunilateral	11	6 months	72
2016	Elias et al. 7	Prospective, multicenter, randomized sham-controlled	Medication-refractory ET	VIM unilateral	76	3 months	47
2018	Chang et al. 10	Prospective	Medication-refractory ET	VIM unilateral	67	6 months	55
2021	Iorio-Morin et al. 13	Prospective, single-arm, single-blinded phase 2	Medication-refractory ET	VIM bilateral (2-staged)	10	3 months	45
2021	Abe et al. 14	Prospective, open-label, multicenter, single-arm confirmatory	Medication-refractory ET	VIMunilateral	35	12M	56
2024	Arcadi et al. 16	Prospective	Medication-refractory ET	VIMunilateral	127	6 months	80
2024	Kaplitt et al. 17	Prospective, open-label, multicenter	Medication-refractory ET	VIMbilateral (2-staged)	51	3 months	66

Abbreviations: ET, essential tremor; HIFU, high-intensity focused ultrasound; VIM, ventral intermediate nucleus of the thalamus.

Note: “2-staged” refers to sequential bilateral procedures.

#### *Long-term*

Prospective randomized controlled trials have demonstrated that unilateral HIFU thalamotomy provided sustained and significant tremor improvement at 3, 4, and 5 years postoperatively, with an overall improvement in QoL measures and no progressive or delayed complications. 11 12 41

Specifically, postural tremor in the treated hand improved by approximately 73% from baseline at both 4 and 5 years posttreatment in the largest prospective, multicenter, long-term follow-up to date. 41 Cosgrove et al. reported that postural tremor severity (CRST Part A) improved by 72.6% at 1 year, 77.0% at 2 years, 76.1% at 3 years, 73.3% at 4 years, and 73.1% at 5 years, with all changes being statistically significant ( *p* < 0.0001 at each time point). 41 Long-term results in ET are summarized in Table 2 .

**Table 2:** Long-term (> 2 years) clinical studies on HIFU thalamotomy targeting the VIM for ET

Year	Author	Study design	Indication	Target	Patients (n)	Follow-up (years)	Tremor improvement (%)
2019	Park et al. 11	Prospective, randomized controlled	Medication-refractory ET	VIMunilateral	15	4	56
2019	Halpern et al. 12	Prospective, controlled, multicenter	Medication-refractory ET	VIMunilateral	23	3	56
2023	Cosgrove et al. 41	Prospective, observational, controlled, multicenter	Medication-refractory ET	VIMunilateral	40	5	73

Abbreviations: ET, essential tremor; HIFU, high-intensity focused ultrasound; VIM, ventral intermediate nucleus of the thalamus.

Moreover, the composite hand tremor and motor function score (CRST Parts A and B) demonstrated a 54.7% reduction at 1-year of follow-up, 56.2% at 2 years, 52.1% at 3 years, 49.5% at 4 years, and 40.4% at 5 years compared with baseline. Functional disability (CRST Part C) also improved significantly, with reductions of 67.4% at 1 year, 60.1% at 2 years, 56.1% at 3 years, 49.0% at 4 years, and 44.5% at 5 years.

Improvements to QoL, as measured by the quality of life in essential tremor questionnaire (QUEST), remained significant across multiple domains, including physical, psychological, and work/finance dimensions, throughout the 5-year follow-up. Importantly, no new serious AEs were reported beyond the 1 ^st^ year, and all persistent AEs at 5 years were classified as mild or moderate in severity. 41

A more recent systematic review and meta-analysis assessed the efficacy and safety of HIFU thalamotomy in patients with ET, providing robust quantitative evidence of its clinical effectiveness. 43 The analysis included SMDs derived from multiple studies. It demonstrated substantial short-term benefits: hand tremor severity improved by an SMD of 3.06 at 1-month, functional disability (CRST Part C) improved by 3.05, and QoL (QUEST) improved by 1.47. These improvements remained strong throughout 12 months, with SMDs of 2.36 (tremor), 2.85 (disability), and 1.41 (QoL), indicating sustained clinical benefit over the 1 ^st^ year postprocedure. Notably, a meta-regression analysis identified a statistically significant decline in treatment efficacy over time ( *p* = 0.0021), with 14.58% of the outcome variability explained by time (R ^2^ = 0.1458), suggesting a gradual attenuation of benefit up to 5 years. 43

Although most patients experience meaningful tremor reduction, a minority may exhibit suboptimal clinical response or gradual attenuation of benefit over time. Long-term follow-up studies and meta-analyses suggest that approximately 5 to 15% of patients may experience insufficient tremor control or recurrence, which may be related to targeting variability, anatomical differences, or disease progression. 7 41 43

### Parkinson's disease

Since the publication of the first prospective, double-blind, sham-controlled, randomized trial, VIM-HIFU has demonstrated significant efficacy in managing refractory tremors in PD (62%) improvement. 8

Recent data further highlight its effectiveness: a cohort of 49 patients undergoing HIFU thalamotomy showed 100% improvement immediately postprocedure, with sustained improvements at 3 months of approximately 83% for rest, 95% for postural, and 100% for intention tremors. 15

When targeting the STN, patients with PD demonstrate significant improvement in motor function in the OFF-medication state at 6 months postprocedure, ranging from 53 to 57.8% as measured by the UPDRS, which showed 71 to 83.5% improvement in rigidity, 37 to 69.4% in bradykinesia, and 77 to 91.5% in tremor control at 6 months postprocedure. 18 21 A randomized, sham-controlled, double-blind, clinical trial confirmed a 52.6% improvement in motor symptoms after 4 months compared with only 4.2% improvement in the control group. 19

For patients whose symptoms progress on the untreated side, staged bilateral HIFU-STN has also been proven safe and effective. The second HIFU-STN procedure improved the contralateral body side by 64.3%. 20 These findings support it as a promising alternative for patients with PD who are not optimal candidates for DBS.

The GPi has also demonstrated positive results in patients with PD experiencing dyskinesias, motor fluctuations, and motor impairment, particularly in the “off” medication state. A significant response to GPi-targeted HIFU was found in 69% of patients at 3 months postprocedure, resulting in a 37% difference in points compared with the control group. 23 Unilateral pallidotomy for the treatment of PD symptoms was approved by the FDA in November 2021.

Gallay et al. 24 evaluated ten patients with medication-refractory PD who underwent bilateral pallidothalamic tractotomy with a 1-year follow-up. The procedure yielded a 52% improvement in total Movement Disorder Society's Unified Parkinson's Disease Rating Scale, Part III (MDS-UPDRS III) motor examination score, including rigidity (67%), bradykinesia (54%), and tremor (91%), while gait and postural stability remained globally unchanged. Speech difficulties increased by 58%, but dyskinesias and dystonia improved markedly, and L-Dopa intake decreased from 690 ± 250 to 110 ± 190 mg. These results demonstrated clinically meaningful and durable bilateral benefits with an acceptable safety profile, contributing to the subsequent FDA regulatory approval of pallidothalamic tractotomy for PD in 2025. 24

Table 3 summarizes the clinical studies on HIFU for PD.

**Table 3:** Clinical studies on HIFU targeting the VIM, STN, GPi ,and PTT for PD

Year	Author	Study design	Indication	Target	Patients (n)	Follow-up (months)	Improvement (%)
2017	Bond et al. 8	Prospective, 2-center, double-blind, sham-controlled, pilot, randomized trial	Medication refractory TdPD	VIMunilateral	27 (20 active, 7 sham)	3	Tremor: 62
2018	Martínez-Fernández et al. 18	Prospective, open-label pilot study	PD with asymmetric motor signs, reluctant or unsuitable for DBS	STNunilateral	10	6	Total MDS-UPDRS III: 53; Rigidity: 71; Bradykinesia: 37; Tremor: 77
2020	Martínez-Fernández et al. 19	Prospective, randomized, sham-controlled, double-blind trial	PD with asymmetric motor signs, reluctant or unsuitable for DBS	STN unilateral	40 (27 active, 13 sham)	4	Total MDS-UPDRS III: 52.6; Rigidity: 60; Bradikynesia: 33; Tremor: 83
2021	Eisenberg et al. 22	Multicenter, open-label trial	PD and levodopa responsiveness, asymmetrical motor signs, and motor fluctuations, including dyskinesias	GPi	20	3	UDysRS: 59–43; Total UPDRS III: 44.5 at 3months
2021	Gallay et al. 24 ,*	Single-center, retrospective observational analysis.	Chronic and therapy-resistant PD (mixed and TdPD)	PTT bilateral	10	12	Total MDS-UPDRS III: 52; Rigidity: 67; Bradykinesia: 54; Tremor: 91
2023	Krishna et al. 23	Multicenter, prospective, double-blind, randomized, sham-controlled trial	PD + dyskinesias or motor fluctuations and motor impairment in the OFF-MED	GPi	94 (69 active, 25 sham)	3	UPDRS III or UDysRS: 69
2023	Chua et al. 15	Prospective, single-center, open-label	Medication refractory TdPD	VIM unilateral	34	3	Tremor: 62
2024	Martínez-Fernández et al. 20	Prospective, open-label, case series, pilot study	PD untreated side had progressed, not optimally controlled with medication	STN bilateral	6	6	Total MDS-UPDRS III: 40,6; Rigidity: 57; Bradykinesia: 68; Tremor 90
2024	Armengou-Garcia et al. 21	Prospective, single-center, open-label	PD with asymmetric motor signs, reluctant or unsuitable for DBS	STN unilateral	20	6	Total MDS-UPDRS III: 78.6; Rigidity: 83.5; Bradykinesia: 69.4; Tremor: 91.5

Abbreviations: DBS, deep brain stimulation; GPi, globus pallidus internus; HIFU, high-intensity focused ultrasound; MDS-UPDRS III, Movement Disorder Society's Unified Parkinson's Disease Rating Scale, Part III (motor examination); OFF-MED, “off” medication state; PD, Parkinson's disease; PTT, pallidothalamic tract; STN, subthalamic nucleus; TdPD, tremor-dominant Parkinson's disease; UPDRS III, Unified Parkinson's Disease Rating Scale, Part III; UDysRS, Unified Dyskinesia Rating Scale; VIM, ventral intermediate nucleus of the thalamus.

Notes: “Active” and “sham” indicate the groups in randomized sham-controlled trials. “Bilateral” refers to 2-staged, sequential contralateral procedures performed > 6 months after the first treatment.

## LEARNING CURVE AND TECHNOLOGY ADVANCES

The HIFU technology relies on team expertise and advanced technology, and it is anticipated that outcomes will improve over time with fewer AEs and increased patient comfort. Recent clinical trials have demonstrated significant advancements in tremor control, reporting improvements of approximately 80% in ET and 91% in PD tremors. 16 21 Enhanced precision in targeting and optimizing sonication parameters may contribute to the observed outcomes. As “technology improves with age”, 44 procedural refinement over time also contributes to enhanced results.

Recent advances in HIFU thalamotomy for ET have markedly improved the precision of lesion targeting and prediction of clinical outcomes, through the integration of high-resolution neuroimaging and tractography-based modeling. Among these innovations, the development of standardized four-tract tractography pipelines—reconstructing the decussating and nondecussating dentatorubrothalamic tracts, corticospinal tract, and medial lemniscus—has been shown to systematically refine indirect targeting by positioning tractography-defined VIM coordinates approximately 1.8 ± 0.88 mm medial–anterior to traditional landmarks. 45 This technique enhances the initial sonication accuracy, enables earlier tremor suppression, and reduces off-target sensory or motor effects.

In a pivotal multicenter study involving 351 patients from three international institutions, Chua et al. 46 identified a specific “sweetspot” within the VIM of the thalamus that, when lesioned, was associated with a mean 86.0% improvement in Fahn-Tolosa-Marin (FTM) tremor scores at 1 year postprocedure in ET patients. This optimal target explained 15% of the variance in clinical outcomes (R ^2^ = 0.15, *p* < 0.005), and its robustness was confirmed by validation in independent cohorts. The study further introduced voxel-wise “sourspot” mapping to delineate lesion zones associated with specific AEs such as sensory deficits from posterior extension into the ventral posterolateral (VPL) and/or posteromedial (VPM) nuclei, contralateral weakness from lateral spread into the internal capsule, and gait imbalance linked to ventrolateral thalamic involvement. 46

Additionally, fiber filtering analyses confirmed that superior tremor control correlated with lesions intersecting the cerebellothalamic tract (CTT), reinforcing the tract-based targeting rationale (peak t = 4.34). Although larger lesion volumes (mean: 462.90 ± 190.15 mm ^3^ ) were associated with greater clinical efficacy, they also conferred an increased risk of AEs, underscoring the therapeutic trade-off between efficacy and safety. 46

As technology advances, specific considerations unique to TdPD are increasingly integrated into treatment planning and target selection. A multicenter, retrospective study evaluated the clinical efficacy and optimal target localization of HIFU thalamotomy in patients with TdPD. 47 The study included 32 patients from three centers and an independent validation cohort of 9 additional patients. Tremor severity, as assessed by hand tremor scores (using CRST and MDS-UPDRS), demonstrated a mean improvement of 55.5 ± 36% at 3 to 12 months postprocedure across the multicenter TdPD cohort. Subgroup analysis showed site-specific improvements ranging from 37.7 to 68.3%, with the independent validation cohort demonstrating a 46.1 ± 18.6% improvement in MDS-UPDRS Part III scores.

Notably, voxel-wise probabilistic efficacy mapping identified that the optimal lesion location for TdPD differed significantly from the traditional ET target, with the peak efficacy located approximately 3.5 mm anterior and 3 mm dorsal to the standard ET target, within the border zone between the VIM and ventral oral posterior (VOP) nuclei. The region of maximal clinical benefit corresponded to areas with distinct structural connectivity projections to the motor and premotor cortices, differing from the connectivity of the ET target with the motor and somatosensory regions. Furthermore, individual clinical outcomes in patients with TdPD could be accurately predicted based on the lesion's overlap with the disorder-specific voxel efficacy map, and these findings were externally validated. 47

## ADVERSE EFFECTS

The AEs are categorized as mild (minor inconvenience, not affecting daily routine activities), moderate (bothersome, interferes with routine daily activities), or severe (incapacitating, cannot perform activities of daily living). Most AEs are mild and transient. 7 8 16 17 The period immediately following the procedure, particularly within the first 30 days, is marked by transient AEs due to lesion edema. 6 The longest follow-up reported for HIFU thalamotomy reported no serious AEs at 5 years. The 4 and 5-year follow-ups reported 71 mild and 29% moderate cases, respectively. 32

The type and severity of these complications depend on the anatomical target, whether thalamotomy, subthalamotomy, pallidotomy, or pallidothalamic tractotomy. The key side effects, categorized by the lesioning target, are outlined in Table 4 .

**Table 4:** Adverse effects associated with HIFU thalamotomy, VIM 2016/2017, VIM 2023/2024, GPi pallidotomy, STN, and PTT

Adverse effect	VIM (2016/2017)	VIM (2023/ 2024)	Adverse effect	GPi	Adverse effect	STN	Adverse effect	PTT
Gait disturbances	Total: 36–40% Persistent: 4–9%	Total: 16–38.24% Persistent: 5–12.77%	Gait disturbances	Total: 2.94% Persistent: 0	Dyskinesia	Total: 15–22% Persistent: 5–11%	Blepharospasm	Total: 10%
Paresthesias (includes finger and facial) or numbness	Total: 27–38% Persistent: 14–19%	Total: 8.82–26.47% Persistent: 17–20%	Dysarthria	Total: 2.94% Persistent: 1.47%	Contralateral weakness	Total: 15–19% Persistent: 7–10%	Hiccups with breathing and speech difficulties	Total: 10%
Dysmetria	Total: 5–12% Persistent: 0–4%	Total: 14.7% Persistent: 0–5%	Dysgeusia	Total: 2.94% Persistent: 0	Dysarthria	Total: 10–26% Persistent: 0–11%	Postoperative falls	Total: 10%
Contralateral weakness	Total: 4–10% Persistent: 2–10%	Total: 2.94–17.65% Persistent: 0–5%	Visual disturbance	Total: 1.47% Persistent: 1.47%	Gait disturbances	Total: 15–48% Persistent: 5–7%	Uncontrollable laughter	Total: 10%
Dysgeusia	–	Total: 5.8–14% Persistent: 5–6.38%	Facial weakness	Total: 1.47% Persistent: 1.47%	Facial weakness	Total: 20% Persistent: 0		
Dysphagia	Total: 2%	Total: 2.94–6% Persistent: 5–6.38%						
Dysarthria	Total: 2–5% Persistent: 5%	Total: 5.88–16% Persistent: 14.89%						

Abbreviations: GPi, globus pallidus internus; HIFU, high-intensity focused ultrasound; PTT, pallidothalamic tract; STN, subthalamic nucleus; VIM, ventral intermediate nucleus.

Notes: For each adverse effect, the table presents the total incidence and the rate of persistent symptoms reported at follow-up in the respective studies. The percentages refer to the proportion of treated patients who developed the adverse effect . “Persistent” refers to adverse effects that remained present ≥ 3 months after the procedure.

In general, AEs related to sonication are mild, transient, and fully resolved by the end of the HIFU procedure. The most frequently reported symptom is headache, which occurs in up to 60% of patients. Other common intraprocedural effects include head discomfort described as heat or pressure (15–30%), vertigo (21–40%), nausea (20%), and neck, back, and shoulder pain (9–20%). Less frequent events included scalp tingling (5–7%), anxiety (5–10%), and vomiting (4%). All these manifestations are considered temporary and self-limited, typically resolving before the end of the sonication session without requiring specific intervention. 7 8

To minimize the risk of side effects during HIFU, low-power sonication of short duration (10–20 seconds) was applied before the actual treatment phase. These preliminary sonications induce subablative temperatures, which are sufficient to visualize thermal changes on MRI thermometry images but remain below the threshold for tissue lesioning. 5 This approach helps accurately position the “hot spot” and enhances the safety of subsequent high-power sonications. Although this strategy improves the safety profile, AEs can still occur, most of which are related to sonication, the stereotactic frame, or the lesion itself. 7 8

## DEEP BRAIN STIMULATION VERSUS HIFU

Firstly, DBS has long been regarded as the gold standard surgical intervention for treating refractory ET and PD. Since its FDA approval for ET in 1997, PD in 2002, and dystonia in 2003, 48 the technique has accumulated extensive evidence supporting its robust and durable efficacy. Longitudinal studies report tremor reductions of approximately 66% in patients with ET after 1 year, decreasing to 48% beyond a decade, while in PD, benefits remain as high as 73% at 1 year and 69% over 10 years. 34 48 However, despite these outcomes, the uptake of DBS is limited. Studies indicate that approximately 45% of patients who qualify for the procedure decline it, often due to apprehension about surgery, cost, device maintenance, or the desire to wait for less invasive options. 49

This treatment involves stereotactic neurosurgical implantation of electrodes, subcutaneous pulse generators, and lead extensions. Invasiveness contributes to a non-negligible AE profile. Surgical complications include intracerebral hemorrhage (3%), subdural hematoma (1.5–1.76%), asymptomatic bleeding (2.9%), cerebrospinal fluid (CSF) leaks (0.76%), mental status changes (3–3.5%), and wound dehiscence (2.6%). 50 51 Device-related complications, reported in 5 to 11.5% of cases, include infections (1.2–3.4%), lead fractures (5.3%), skin erosion (2.5%), and lead misplacement or migration necessitating repositioning (3.8%). 50 51

A retrospective review of over 500 DBS cases for ET, PD, and dystonia over a 10-year period found consistent complication rates across targets, including the STN, GPi, and VIM. 51 Beyond surgical and hardware risks, stimulation-induced side effects are common in VIM-DBS for ET, ranging from 26 to nearly 50% in the 1 ^st^ year, 34 50 often requiring several reprogramming sessions. Paresthesia and dysarthria are among the most frequently reported AEs, occurring in approximately 17% of patients, particularly those with VIM targeting. Other notable side effects include gait disturbances and ataxia in 10% of cases, as well as hemiparesis and persistent headache in rare instances. 34 52

Although stimulation-related effects are usually transient and adjustable, they may limit the ability to optimize the therapeutic program. Moreover, device maintenance, including impedance checks, battery replacements (every 4–5 years), and continued follow-up 3 times per year, is usually necessary to maintain functionality and ensure tolerability. 53

Second, HIFU emerged as a minimally invasive alternative treatment that delivers immediate tremor relief without the need for surgical incisions or implants. The procedure is performed under real-time MRI thermography and is typically completed in a single session, with patients being discharged the same day or within 24 hours. Recovery is rapid, and device programming, hardware maintenance, or future surgeries are not required. As such, HIFU completely avoids hardware- and surgery-related complications, which may explain its growing appeal, particularly among patients who are ineligible for or averse to invasive procedures. 49

Although the overall safety profile of HIFU is favorable, it is not without risk, as mentioned above. Notably, long-term follow-up has reported no new AEs beyond 12 months posttreatment, and no severe complications have been documented after 5 years, indicating an excellent and sustained safety profile. However, the irreversible nature of these lesions means that AEs, if present, may not be modifiable. Furthermore, a small percentage of patients experience partial loss of benefit between 6 and 12 months, 15 underscoring the need for precise targeting. Still, in cases where symptom progression or recurrence occurs, DBS electrodes have been safely implanted in previously lesioned areas up to 4 years after HIFU, demonstrating the viability of a sequential or complementary strategy. 17 21 23 41

In a contemporary comparison 54 of unilateral thalamic DBS and HIFU thalamotomy for medication-refractory ET, both interventions demonstrated significant and sustained tremor reduction at 1-year of follow-up. The DBS cohort exhibited an average 80% improvement in postural tremor severity, as measured by the CRST, whereas the HIFU group achieved a 68% reduction. Although DBS patients presented with more severe baseline tremor scores (CRST: 3.1 versus 2.1 in HIFU), posttreatment tremor severity scores were statistically comparable between the two groups at all follow-up points (3, 6, and 12 months). Improvements to QoL were also observed in both cohorts, with HIFU showing a 49% improvement in QUEST scores and DBS demonstrating a 37% improvement, although direct statistical comparisons were limited due to differences in baseline characteristics and reporting methodologies. 54

Cost considerations are also relevant when comparing HIFU and DBS. Although HIFU avoids implanted hardware and long-term device maintenance, the procedure requires specialized equipment and infrastructure, leading to substantial upfront costs. In contrast, DBS involves expenses related to electrode implantation, pulse generators, and ongoing device management, including programming and eventual hardware replacement. 55 56 However, the overall costs of both approaches may become comparable in high-income healthcare systems, reflecting higher initial procedural costs for HIFU and greater long-term maintenance costs for DBS. Cost-effectiveness analyses have shown favorable economic profiles for HIFU.

In ET, HIFU demonstrated higher utility gains and lower projected costs than DBS. 57 Similar findings have been reported in TdPD, where HIFU remained cost-effective compared with medical therapy and economically competitive with DBS despite slightly lower gains in quality-adjusted life years (QALYs), a measure that integrates survival and health-related QoL. 58 Access to HIFU nevertheless remains limited in many regions due to the need for dedicated equipment and institutional expertise, whereas DBS has been more widely adopted across neurosurgical centers worldwide. 55

Before the development of DBS and HIFU, traditional ablative procedures such as radiofrequency thalamotomy and pallidotomy were widely used to treat tremor and parkinsonian symptoms. 56 59 Radiofrequency thalamotomy can achieve tremor reductions of approximately 70 to 90% in ET, although bilateral procedures were historically limited by higher rates of dysarthria and gait disturbances. 55 Compared with conventional lesioning, HIFU offers the advantage of being incisionless and enabling real-time MRI thermometry during lesion creation, improving targeting precision and safety. 1 32 Nevertheless, radiofrequency procedures remain less costly and more widely available in many low- and middle-income settings.

In summary, DBS offers adjustable, long-term symptom control but requires surgical implantation and regular maintenance and carries a relatively high burden of both procedural and stimulation-related risks. Meanwhile, HIFU provides immediate, durable tremor relief with a minimally invasive approach and minimal follow-up; however, it lacks the flexibility of parameter modulation and is subject to the inherent limitations of lesion-based therapies. For selected patients—particularly those with unilateral symptoms, comorbidities precluding surgery, or those living in remote areas—HIFU represents a powerful alternative. As technology evolves and targeting becomes increasingly precise, both therapies should be viewed as complementary modalities in the treatment of movement disorders, with clinical decision-making driven by individual risk profiles, anatomical targeting, and patient preference.

Table 5 summarizes the main distinctions between DBS and HIFU in terms of targets, procedural characteristics, target confirmation methods, intraoperative considerations, time to clinical benefit, efficacy, AEs, reversibility, recovery time, follow-up needs, and factors associated with procedural success.

**Table 5:** Key differences between DBS and MR-guided HIFU

	DBS	HIFU
**Target**	VIM, PSA, STN, GPi	VIM, STN, GPi, PTT
**Approach**	Invasive, requires skin and skull opening, no need for full head shaving	Incisionless, full head shaving required
**Target confirmation**	Awake MER, clinical evaluation, LFPs, MRI-guided	Real-time MRI temperature imaging and evaluation
**Intraprocedural interference**	Brain shifting due to CSF leak	No
**Time to onset of effect**	Improvement after first programming session, 3–6 months to obtain optimal results	Immediate improvement
**Efficacy**	High efficacy Tremor reduction: ∼50–75% in ET ∼50–70% in TdPD	High efficacy Tremor reduction: ∼50–75% in ET ∼60–90% in TdPD
**Adverse effects (AEs)**	∼3% risk of infection, intracerebral hemorrhage, hardware issues ∼25% stimulation-related AEs, mild or reversible with programming adjustments	No “device”-related AEs, Risk include side effects that can be irreversible
**Reversibility**	Partially reversible, can be turned off	Irreversible, permanent tissue ablation
**Recovery time**	Longer recovery post-surgery	Shorter recovery; quick return to routine
**Follow-up care**	Regular follow-ups for device adjustment, impedance check and battery check	Minimal follow-up post initial recovery
**Procedure success**	Correlates with surgical team experience, learning curve	Correlates with surgical team experience, learning curve

Abbreviations: AE, adverse effect; CSF, cerebrospinal fluid; DBS, deep brain stimulation; ET, essential tremor; GPi, globus pallidus internus; HIFU, high-intensity focused ultrasound; LFP, local field potential; MER, microelectrode recording; MRI, magnetic resonance imaging; PSA, posterior subthalamic area; PTT, pallidothalamic tract; STN, subthalamic nucleus; TdPD, tremor-dominant Parkinson's disease; VIM, ventral intermediate nucleus.

In conclusion, HIFU has emerged as an important minimally invasive therapeutic option for patients with medication-refractory ET and PD. The available evidence demonstrates substantial tremor reduction with VIM thalamotomy and meaningful motor improvement when other targets, such as the STN, GPi, and pallidothalamic tract, are selected according to the clinical phenotype. This highlights the importance of appropriate target selection based on the predominant clinical manifestations.

While clinical teams continue to refine their techniques through cumulative experience, HIFU is poised to complement rather than compete with established therapies, such as DBS, fostering a multidisciplinary approach that prioritizes individualized care and expands treatment options for patients with movement disorders.

Future technological advances, including tractography-based targeting, AI-assisted thermography, and predictive modeling of lesion characteristics, may further improve targeting precision and procedural outcomes. 60 These refinements, coupled with a deeper understanding of anatomical “sweet spots,” are expected to support more accurate patient selection and optimize the therapeutic application of HIFU in ET and PD.
